# Super-Multiple Deletion Analysis of Type III Effectors in *Ralstonia solanacearum* OE1-1 for Full Virulence Toward Host Plants

**DOI:** 10.3389/fmicb.2020.01683

**Published:** 2020-07-30

**Authors:** Ni Lei, Li Chen, Akinori Kiba, Yasufumi Hikichi, Yong Zhang, Kouhei Ohnishi

**Affiliations:** ^1^The United Graduate School of Agricultural Sciences, Ehime University, Matsuyama, Japan; ^2^College of Food Engineering and Nutritional Science, Shaanxi Normal University, Xi’an, China; ^3^Faculty of Agriculture and Marine Science, Kochi University, Kochi, Japan; ^4^College of Resources and Environment, Southwest University, Chongqing, China; ^5^Interdisciplinary Research Center for Agriculture Green Development in Yangtze River Basin, Southwest University, Chongqing, China; ^6^Research Institute of Molecular Genetics, Kochi University, Kochi, Japan

**Keywords:** *Ralstonia solanacearum*, type III effector, multiple deletion, pathogenesis, core effectors

## Abstract

*Ralstonia solanacearum* species complex (RSSC) posses extremely abundant type III effectors (T3Es) that are translocated into plant cells via a syringe-like apparatus assembled by a type III secretion system (T3SS) to subvert host defense initiated by innate immunity. More than 100 T3Es are predicted among different RSSC strains, with an average of about 70 T3Es in each strain. Among them, 32 T3Es are found to be conserved among the RSSC and hence called the core T3Es. Here, we genetically characterized contribution of abundant T3Es to virulence of a Japanese RSSC strain OE1-1 toward host plants. While all the T3Es members of AWR family contributed slightly to virulence, those of the GALA, HLK, and SKWP families did not influence full virulence of OE1-1. Mutant OE1-1D21E (with deletion of all 21 T3Es members of four families) exhibited slightly impaired virulence, while mutant OE1-1D36E (deleting all 21 T3Es of 4 families and 15 core T3Es) exhibited substantially reduced virulence. Mutant OE1-1D42E (deleting all 21 T3Es of 4 families, 15 core T3Es and 6 extended core T3Es) failed to cause any disease on tobacco plants with leaf infiltration but retained faint virulence on tobacco plants with petiole inoculation. The proliferation of mutant OE1-1D42E in tobacco stems was substantially impaired with about three orders of magnitude less than that of OE1-1, while no impact in tobacco leaves if directly infiltrated into leaves. On the contrary, the OE1-1D42E mutant retained faint virulence on eggplants with leaf infiltration but completely lost virulence on eggplants with root-cutting inoculation. The proliferation of OE1-1D42E mutant both in eggplant leaves and stems was substantially impaired. Intriguingly, mutant OE1-1D42E still caused necrotic lesions in tobacco and eggplant leaves, indicating that some other than the 42 removed effectors are involved in expansion of necrotic lesions in host leaves. All taken together, we here genetically demonstrated that all the core and extended core T3Es are nearly crucial for virulence of OE1-1 toward host plants and provided currently a kind of T3Es-free strain that enables primary functional studies of individual T3Es in host cells.

## Introduction

*Ralstonia solanacearum* is a soil-borne Gram-negative plant vascular bacterium that is widely distributed and can cause lethal bacterial wilt on more than 450 plant species belonging to 50 botanical families worldwide (Mansfield et al., 2012; Jiang et al., 2017). Like in many pathogenic bacteria of animals and plants, a syringe-like type III secretion system (T3SS) is one of the essential pathogenicity determinants in *R. solanacearum* that is a translocation apparatus and ensures direct translocation of virulence proteins (called type III effectors, T3Es) into plant cell cytosol to subvert host defense initiated by innate immunity (Poueymiro and Genin, 2009; Genin, 2010). *R. solanacearum* strains are currently referred to as a *Ralstonia solanacearum* species complex (RSSC) due to their extreme heterogeneity, while the T3SS is considerably conserved among the RSSC (Genin and Denny, 2012; Coll and Valls, 2013). Different from the T3SS, T3Es are hugely variable among different RSSC strains (Mukaihara et al., 2010; Peeters et al., 2013; Sabbagh et al., 2019). There are more than 100 T3Es predicated among different RSSC strains with an average of about 70 T3Es per strain that is significantly more abundant than those of any other plant pathogenic bacteria (less than 30 T3Es in each plant pathogenic bacterium, such as *Pseudomonas syringae* or the *Xanthomonads*) (Sébastien et al., 2011; Genin and Denny, 2012; Peeters et al., 2013). Abundant studies have now demonstrated that highly variable repertoires of T3Es might be responsible for the host range of different RSSC strains (Genin and Denny, 2012; Coll and Valls, 2013). Two RSSC strains of GMI1000 and RS1000 are virulent on tomato plants, and they are avirulent on tobacco plants that elicit a hypersensitive response (HR) in tobacco leaves. Two strains have almost identical repertoires of T3Es that comprise 72 and 74 T3Es, respectively (Mukaihara et al., 2010; Peeters et al., 2013). Deletion of T3Es of both RipAA and RipP1 enables GMI1000 to cause wilt symptoms on tobacco plants (Poueymiro et al., 2009), while removal of RipB enables RS1000 to cause wilt symptoms on tobacco plants, and RipAA and RipP1 contribute in part to avirulence of RS1000 on tobacco plants (Nakano and Mukaihara, 2019), indicating that different effector repertories are recognized by different plants, which ultimately leads to virulence or avirulence of the pathogen.

**FIGURE 1:**
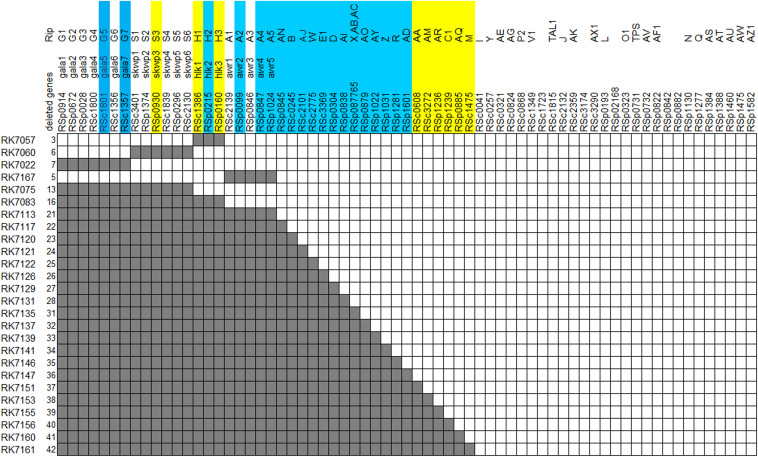
Diagram of effector deletion mutants. Deleted genes are marked in gray. Core and extended core effectors are shaded by blue and yellow, respectively.

A feature of T3Es repertoires in the RSSC is the existence of functional redundancy with several multigenic families, including the AWR (RipA in a unified nomenclature, five members), GALA (RipG, seven members), HLK (RipH, three members), PopP (RipP, two members) and SKWP (RipS, six members) (Peeters et al., 2013; Sabbagh et al., 2019). Abundant studies have been to date carried out on these multigenic families that are collectively, but not individually, required for full virulence of RSSC strains toward host plants (Angot et al., 2006; Remigi et al., 2011; Solé et al., 2012). Comparing T3Es of 11 sequenced strains representative of four RSSC phylotypes demonstrates that 22 T3Es are conserved among all 11 RSSC strains and are assigned as the core T3Es. 10 T3Es are conserved among 10 out of 11 RSSC strains and are designated as the additional core T3Es (Peeters et al., 2013). We refer to the additional core as the extended core in this study. Note that four multigenic families of the AWR, GALA, HLK, and SKWP contain nine core and extended core T3Es. In addition to T3Es of above four multigenic families, more and more individual T3Es are currently demonstrated to be functional for *R. solanacearum* to invade host cells (Nakano and Mukaihara, 2018, 2019; Sun et al., 2019; Zhuo et al., 2020). We recently announced complete genome sequence of OE1-1 (chromosome: NZ_CP009764.1, megaplasmid: NZ_CP009763.1) that was initially isolated from eggplant in Japan. Both OE1-1 and GMI1000 belong to phylotype I, sharing more than 99% of genomic similarity (data not shown), while OE1-1 is virulent in both tomato and tobacco plants (Kanda et al., 2003). Blast search analysis indicates that OE1-1 possesses more than 70 T3Es, including 21 T3E members of above 4 multigenic families. Generally, OE1-1 contains 21 core T3Es and 9 extended core T3Es (Figure 1).

To date, several hundred papers have focused on a either few or single T3Es to decipher their functional roles in host cells that mainly focus on molecular interaction with host innate immunity, such as manipulating host proteasome, eliciting host cell death, triggering expression of plant genes, and/or displaying biochemical activities on plant protein targets (Deslandes and Genin, 2014). There are a limited number of studies focusing on coordinate contribution of abundant T3Es to the infection process of RSSC strains toward host plants. We currently characterized coordinated contribution of each T3Es family to full virulence of OE1-1 and demonstrated that one or more T3Es of the GALA family, SKWP family, or HLK family did not contribute to virulence of OE1-1 toward host plants. All the T3Es members of the GALA family or HLK family slightly contribute to full virulence of OE1-1 toward host tomato and tobacco plants, which is consistent with previous reports in other RSSC strains (Remigi et al., 2011; Solé et al., 2012; Chen et al., 2013, 2018). We here focused on dozens of T3Es (over 40 T3Es including the core and extended core T3Es) to clarify their joint contribution to full virulence of OE1-1 toward host plants.

## Materials and Methods

### Bacterial Strains and Culture Conditions

*R. solanacearum* T3Es mutants used in this study (listed in Supplementary Table S1 and Figure 1) are derivatives of OE1-1, which is virulent against tomato, tobacco, and eggplants (Kanda et al., 2003). *R. solanacearum* strains were grown at 28°C in nutrient-rich medium containing casamino acids, peptone, yeast extract, and glucose (Boucher et al., 1985). *Escherichia coli* strains of DH12S and S17-1 were grown in Luria-Bertani (LB) medium at 37°C for plasmid construction and conjugational transfer, respectively.

### Mutants Generation With an In-Frame Deletion of Target Genes

In the present study, target genes were in-frame deleted with the pK18mobsacB-based homologous recombination as previously described (Zhang et al., 2018), and many genes could be removed one by one with this system. In brief, two DNA fragments flanking a target gene were conjugated with joint PCR and cloned into pK18mobsacB to generate the desired plasmid (Supplementary Table S1). After validated sequences, each plasmid was individually transferred into OE1-1 or its derivate T3Es mutants by conjugation with *E. coli* S17-1 to generate desired mutants that were confirmed by colony PCR with respective primer pairs (Supplementary Table S1).

### Virulence Assay and Necrotic Lesion Test

Virulence assay was performed in two wilt-susceptible plants of tobacco (*Nicotiana benthamiana*) and eggplant (*Solanum melongena* cv. Senryo-nigou) as previously described (Zhang et al., 2018). *R. solanacearum* cells were inoculated to testing plants cultivated in a culture room at 25°C for 3–4 weeks with two methods of root-cutting and leaf-infiltration. The first method is a more natural way, and the second one allows cells to invade intercellular spaces of leaves directly. Each assay was performed with four biological replicates using 12 plants per trail. Wilt symptoms of plants were daily inspected as a 1–4 disease index. Statistical significance was assessed using a *t*-test or a Tukey-Kramer.

Necrotic lesions were observed in leaves of tobacco and eggplant with leaf infiltration. Briefly, bacterial cells at an OD_600_ of 0.1 (ca. 50 μl) were infiltrated into plant leaves, and the development of necrotic lesions was recorded periodically. Each test was performed with four biological replicates using four plants per trail.

### The *in planta* Bacterial Proliferation Assay

In the present study, the *in planta* bacterial proliferation assay was performed both in leaves and stems of tobacco and eggplant as previously described (Zhang et al., 2019). For proliferation in leaves, bacterial cells at 10^6^ cfu ml^–1^ (ca. 50 μl) were infiltrated into plant leaves, and leaf disks were punched periodically for quantification of cell densities. For proliferation in stems, 2 μl of a bacterial suspension at 10^7^ cfu ml^–1^ was dropped onto the fresh-cut surface of petioles, and cells in stems were harvested periodically for quantification of cell densities. Cell densities were quantified by dilution plating and that in leaves and stems was expressed in cfu cm^–2^ and cfu g^–1^, respectively. Each assay was performed with four biological replicates using six plants per trial. Statistical significance was assessed using a *t*-test.

## Results

### All the T3Es Members of GALA, SKWP, HLK, and AWR Families Contribute Together but in Part to Full Virulence of OE1-1 Toward Tobacco Plants

**FIGURE 2:**
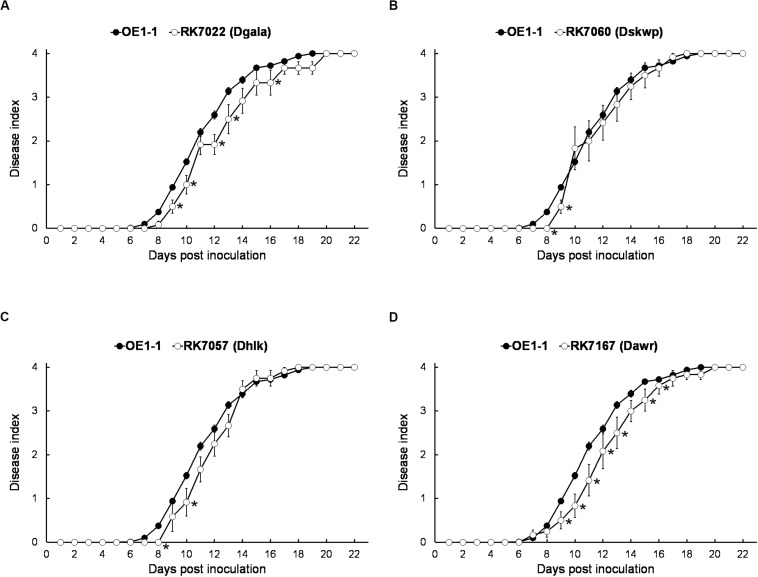
Virulence assay of *R. solanacearum* mutants with T3Es deletion of single-family on tobacco plants with leaf infiltration. Virulence of OE1-1 (the wild type strain) was compared with that of **(A)** RK7022 (D*gala*), **(B)** RK7060 (D*skwp*), **(C)** RK7057 (D*hlk*), and **(D)** RK7167 (D*awr*). About 50 μl of a bacterial suspension at an OD_600_ of 0.1 was infiltrated into *N. benthamiana* leaves with a blunt-end syringe. Wilt symptoms were inspected daily and scored on a disease index scale from 0 to 4 (0, no wilting; 1, 1–25% wilting; 2, 26–50% wilting; 3, 51–75% wilting; and 4, 76–100% wilted or dead). Each assay was repeated with at least four biological replicates using 12 plants per trial. The mean values of all experiments were averaged with standard error (SE). A *t*-test was conducted each day, and asterisks indicated a significant difference (*p* < 0.05).

We generated a series of mutants with deletion of single T3Es, while all of them showed quite similar virulence as OE1-1 on tobacco plants (data not shown). The *gala* mutant RK7022 (deletion of all 7 T3Es of GALA family, D*gala*) and *hlk* mutant RK7057 (deletion of all 3 T3Es of HLk family, D*hlk*) exhibited similar virulence as the wild type OE1-1 on tobacco plants with leaf infiltration (Figures 2A,C; Chen et al., 2013, 2018). The *skwp* mutant RK7060 (deletion of all 6 T3Es of the SKWP family, D*skwp*) also showed similar virulence as OE1-1 (Figure 2B), while the *awr* mutant RK7167 (deletion of all 5 T3Es of AWR family, D*awr*) is substantially less virulent than OE1-1 on tobacco plants with leaf infiltration (Figure 2D). As a control, the T3SS deficient mutant RK5438 (Δ*hrcV*) did not show any virulence on tobacco plants (data not shown; Vasse et al., 2000).

**FIGURE 3:**
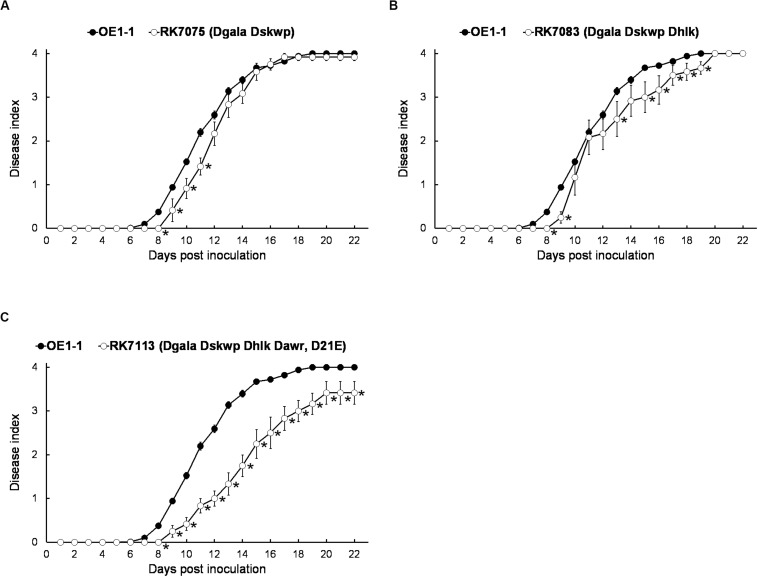
Virulence assay of *R. solanacearum* T3Es mutants with combined deletion of four families in tobacco plants with leaf infiltration. Virulence of OE1-1 was compared with that of **(A)** RK7075 (D*gala* D*skwp*, OE1-1D13E), **(B)** RK7083 (D*gala* D*skwp* D*hlk*, OE1-1D16E), and **(C)** RK7113 (D*gala* D*skwp* D*hlk* D*awr*, OE1-1D21E). Wilt symptoms were inspected daily and scored on a disease index scale from 0 to 4. Each assay was repeated with at least four biological replicates using 12 plants per trial. The mean values of all experiments were averaged with SE. A *t*-test was conducted each day, and asterisks indicated a significant difference (*p* < 0.05).

We, therefore, generated a series of mutants with combined deletion of T3Es from 4 families, RK7075 (D*gala* D*skwp*, deleting 13 T3Es, OE1-1D13E), RK7083 (D*gala* D*skwp* D*hlk*, OE1-1D16E) and RK7113 (D*gala* D*skwp* D*hlk* D*awr*, OE1-1D21E), to evaluate their combined contribution to full virulence of OE1-1 on tobacco plants. Intriguingly, RK7075, and RK7083 exhibited similar virulence as OE1-1 on tobacco plants with leaf infiltration (Figures 3A,B). In contrast, RK7113 showed significantly less virulence than OE1-1 on tobacco plants with leaf infiltration (Figure 3C). All together, all the T3Es members of 4 families contribute together, but partially, to full virulence of OE1-1 toward tobacco plants. These results indicated that some other T3Es besides these four families might be required for full virulence of OE1-1.

### All the Core and Extended Core T3Es Are Jointly Crucial for Virulence of OE1-1 on Tobacco Plants With Leaf Infiltration

**FIGURE 4:**
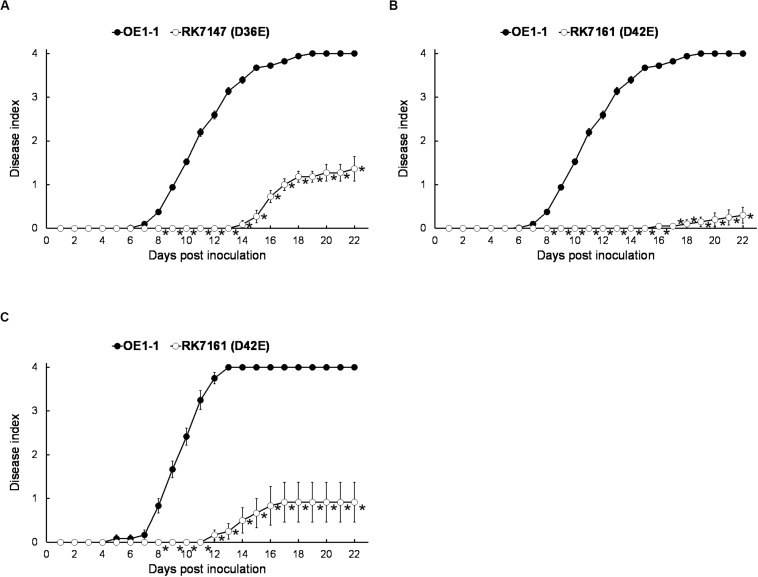
Virulence assay of *R. solanacearum* T3Es mutants with deletion of more core and extended T3Es. OE1-1; RK7147 (D*gala* D*skwp* D*hlk* D*awr*, deletion of 15 more core T3Es, OE1-1D36E); RK7161 (D*gala* D*skwp* D*hlk* D*awr*, deletion of 15 core and six more extended T3Es deletion, OE1-1D42E) were used. Bacterial cells were infiltrated into **(A,B)** tobacco and **(C)** eggplants (*S. melongena* cv. Senryo-nigou). Wilt symptoms were inspected daily and scored on a disease index scale from 0 to 4. Each assay was repeated with at least four biological replicates using 12 plants per trial. The mean values of all experiments were averaged with SE. A *t*-test was conducted each day, and asterisks indicated a significant difference (*p* < 0.05).

Although T3E members of four families of the AWR, GALA, SKWP, and HLK have been functionally validated to be able to alter pathogenic behavior of some RSSC strains on specific hosts (Deslandes and Genin, 2014), RK7113 (OE1-1D21E) was slightly less virulent than OE1-1 on tobacco plants. These four families contain six core T3Es (Peeters et al., 2013), and OE1-1 possesses another 15 core T3Es (Figure 1). We further deleted all these 15 core T3Es one by one from RK7113 (OE1-1D21E) to clarify whether these core T3Es contribute together to full virulence of OE1-1. As expected, mutants deleting more T3Es exhibited less virulence than those with less T3Es deletion on tobacco plants. For instance, mutants of RK7135 (OE1-1D31E) and RK7137 (OE1-1D32E) were less virulent than mutants of RK7126 (OE1-1D26E), RK7129 (OE1-1D27E) and RK7131 (OE1-1D28E) on tobacco plants (Supplementary Figure S1). Mutants of RK7146 (OE1-1D35E) and RK7147 (OE1-1D36E) were much less virulent than mutants of RK7139 (OE1-1D33E) and RK7141 (OE1-1D34E) (Supplementary Figure S1). All the core T3Es were deleted in RK7147 (OE1-1D36E, deletion of 15 more core T3Es from RK7113), which indeed exhibited substantially impaired virulence on tobacco plants (Figure 4A), while it retained faint virulence to some extent since RK7147-inoculated tobacco plants started wilting at 15 days post- inoculation (dpi) and reached disease index one at about 22 dpi (Figure 4A).

OE1-1 possesses 6 extended core T3Es (Figure 1), and we further deleted these 6 T3Es one by one from RK7147 (OE1-1D36E) to clarify whether full virulence of OE1-1 was determined by all the core and extended core T3Es. Mutants of RK7155 (OE1-1D39E) and RK7156 (OE1-1D40E) were less virulent than mutants of RK7151 (OE1-1D37E) and RK7153 (OE1-1D38E) (Supplementary Figure S2). Mutants of RK7160 (OE1-1D41E) and RK7161 (OE1-1D42E, deletion of all 6 extended T3Es from RK7147) failed to cause any wilting symptoms on tobacco plants with leaf infiltration (Figure 4B), confirming that all the core and extended core T3Es are crucial for virulence of OE1-1 on tobacco plants. Different from that with inoculation method of leaf infiltration, RK7161 (OE1-1D42E) caused slight wilting symptoms on eggplants with the petiole inoculation, which enables direct invasion into xylem vessels (Figure 4C). RK7161-inoculated eggplants with petiole inoculation started wilting at 12 dpi and reached disease index one at 22 dpi (Figure 4C), indicating that some other T3Es of OE1-1 besides the core and extended core T3Es could function, especially in xylem vessels of tobacco plants.

### Removal of 42 T3Es From OE1-1 Does Not Eliminate the Formation of Necrotic Lesions in Tobacco and Eggplant Leaves

**FIGURE 5:**
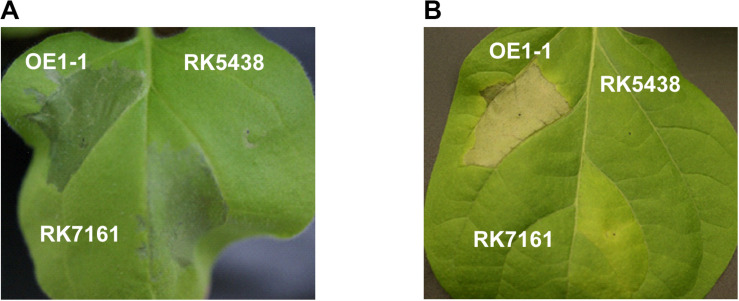
Symptoms of bacterial infiltrated leaves. OE1-1, RK5438 (OE1-1 Δ*hrcV*), and RK7161 (OE1-1D42E) were infiltrated into leaves of **(A)** tobacco and **(B)** eggplant. Approximately 50 μl of a bacterial suspension at an OD_600_ of 0.1 was infiltrated into leaves, and the development of necrotic lesions was observed daily. The representative results are presented, and similar results were obtained in all experiments.

The T3SS is essential for *R. solanacearum* to inject abundant T3Es into host cell cytosol that subverts host defense, and T3SS deficient mutants are well known to fail to cause any wilting symptoms in host plants. For instance, GMI1000 elicits HR in non-host tobacco leaves within 24 h post infiltration (hpi), while its *hrcV* mutant fails to elicit HR (Poueymiro et al., 2009). OE1-1 caused necrotic lesions in tobacco leaves at about 3 dpi and then started to wilt tobacco leaves at about 6 dpi. The *hrcV* mutant of OE1-1, RK5438, failed to cause any necrotic lesions in tobacco leaves (Figure 5A). Intriguingly, RK7161 (OE1-1D42E) caused necrotic lesions in tobacco leaves at about 4 dpi that was about 1-day delayed than OE1-1 (Figure 5A). This phenomenon was also observed in eggplant leaves at about 6 dpi (Figure 5B), indicating that all the core and extended core T3Es are not required for OE1-1 to cause necrotic lesions in host leaves. Some other T3Es besides the 42 removed effectors of OE1-1 might be required to expand necrotic lesions in host leaves.

### All the Core and Extended Core T3Es Are Jointly Crucial for Virulence of OE1-1 in Eggplants

**FIGURE 6:**
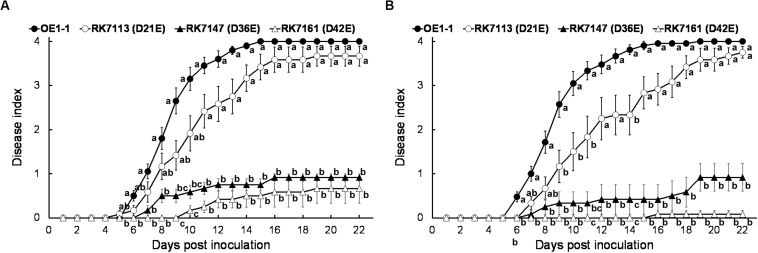
Virulence assay of *R. solanacearum* T3Es mutants in eggplants. Bacterial cells were inoculated with methods of **(A)** leaf infiltration and **(B)** root-cutting inoculation. OE1-1; RK7113 (OE1-1D21E); RK7147 (OE1-1D36E); RK7161 (OE1-1D42E). For leaf infiltration, approximately 50 μl of a bacterial suspension at an OD_600_ of 0.1 was infiltrated into leaves. For root-cutting inoculation, a bacterial suspension was poured onto the root wounded plants at a final concentration of 10^6^ cfu g^– 1^ soil. Wilt symptoms were inspected daily and scored on a disease index scale from 0 to 4. Each assay was repeated with at least four biological replicates using 12 plants per trial. The mean values of all experiments were averaged with SE. A Tukey-Kramer test was conducted each day, and the alphabets indicated significant differences (*p* < 0.05).

RK7161 (OE1-1D42E) caused necrotic lesions in tobacco and eggplant leaves, and we assessed whether RK7161 could cause wilting disease on eggplants. RK7147 (OE1-1D36E) and RK7161 exhibited significantly less virulence on eggplants with leaf infiltration (Figure 6A), which were similar to those on tobacco plants. When eggplants were challenged with a more natural inoculation method of the root-cutting, RK7147 exhibited significantly less virulence, while RK7161 failed to cause any wilting symptoms on eggplants (Figure 6B). Eggplants inoculated with RK5438, the *hrcV* mutant were healthy throughout the experiments (data not shown).

### The Core and Extended Core T3Es Are Essential for OE1-1 to Multiply Both in Leaved and Xylem Vessels of Eggplants, but Only in Xylem Vessels of Tobacco Plants

**FIGURE 7:**
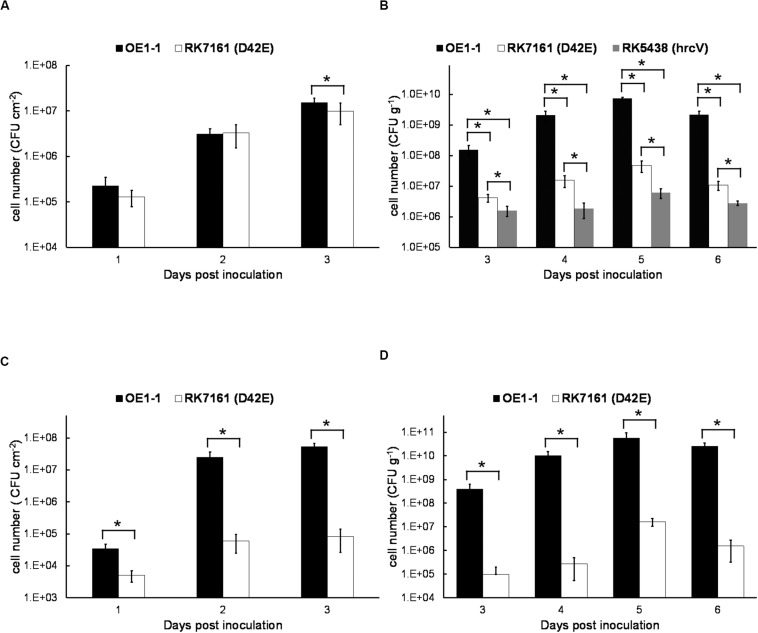
The *in planta* proliferation of bacterial cells. For cell growth in leaves, a bacterial suspension at 10^6^ cfu ml^–1^ was infiltrated into leaves of **(A)** tobacco and **(C)** eggplant with a blunt-end syringe, and leaf disks (0.38 cm^2^) were daily punched for quantification of cell densities by dilution plating. For cell growth in stems, 2 μl of a bacterial suspension at a density of 10^7^ cfu ml^– 1^ was dropped onto a freshly cut petiole surface of **(B)** tobacco and **(D)** eggplant. The stem species (1 cm in length) were daily cut for quantification of cell densities by dilution plating. Black bars, white bars, and gray bars indicate cell growth of OE1-1, RK7161 (OE1-1D42E), and RK5438 (Δ*hrcV*), respectively. Each assay was carried out at least three times. The mean values of all experiments were averaged with SE. A *t*-test was conducted each day, and asterisks indicated a significant difference (*p* < 0.05).

Extensive proliferation in host plants is another essential pathogenicity determinants of *R. solanacearum.* We thus assessed whether the core and extended core T3Es are required for *in planta* proliferation. Tobacco leaves were infiltrated with the bacterial suspension at a concentration of 10^6^ cfu ml^–1^, and cell growth in tobacco leaves was daily assessed until 3 dpi when tobacco leaves became withered and dried. The wild-type strain OE1-1 and RK7161 (OE1-1D42E) grew almost the same ways in tobacco leaves from 1 to 3 dpi (Figure 7A), indicating that these core and extended core T3Es are independent for the intercellular proliferation of OE1-1 at least in tobacco leaves.

We also assessed cell proliferation in xylem vessels of tobacco plants. Tobacco plants were inculcated by dropping a bacterial suspension onto the fresh-cut surface of petioles, and stem pieces were daily harvested for quantification of cell densities. OE1-1 reached to approximately 10^8^ cfu g^–1^ at 3 dpi and got to a maximum of about 10^10^ cfu g^–1^ at 5–6 dpi (Figure 7B). RK7161 grew slowly to a maximum of 5 × 10^7^ cfu g^–1^ at 5–6 dpi. The T3SS deficient *hrcV* mutant, RK5438, did not grow in xylem vessels (Figure 7B). These results confirmed that all the core and extended core T3Es are important for OE1-1 to multiply in tobacco xylem vessels.

Proliferation assay of RK7161 was also performed both in leaves and xylem vessels of eggplants. When directly infiltrated into eggplants leaves, RK7161 grew slowly to the maximum of about 10^5^ cfu cm^–2^ at 3 dpi (Figure 7C), which is about three orders less than that of OE1-1. When directly invaded xylem vessels of eggplants, RK7161 grew slowly to the maximum of 10^7^ cfu g^–1^ at 5 dpi, which was about four orders less than OE1-1 (Figure 7D), confirming that all the core and extended core T3Es are important for OE1-1 to multiply both in the intercellular spaces and xylems of eggplants.

## Discussion

In the present study, we provided genetic evidence to demonstrate that all the core and extended core T3Es are together crucial for virulence of OE1-1 on host tobacco plants. Dozens of T3Es have been confirmed to individually manipulate plant cellular functions and alter plant signal transduction, which results in extensive proliferation of RSSC strains in host plants and gives rise to disease development on host plants. At the same time, some T3Es can be detected by intracellular receptors in non-host plants that activate the immune response to effectively suppress the proliferation of RSSC strains in non-host plants and prevent disease development (Deslandes and Genin, 2014; Macho, 2016). Only limited numbers of studies are focused on the coordinate contribution of abundant T3Es to virulence of the RSSC. Our results demonstrated that mutants with increasing deletion numbers of T3Es exhibited gradually impaired virulence on host tobacco plants (Supplementary Figures S1,S2).

T3Es repertoires are greatly diversified among different RSSC strains, and 32 T3Es are proposed to compose the core and extended core T3Es, which are detected in most of the RSSC strains with genomic searching and probably present in an ancestral *R. solanacearum* strain (Peeters et al., 2013). Our results indeed confirmed that all these core and extended core T3Es play an essential role on virulence of OE1-1 since the mutant deleting all the core and extended core T3Es lost virulence toward host plants. The RSSC has a vast host range, with more than 450 plant species belonging to 50 botanical families. The core and extended core T3Es can probably explain the great adaption of the RSSC to a wide broad range of host species, although there are no apparent clues on T3Es with host specificity determinants (Coll and Valls, 2013; Peeters et al., 2013). Based on unique protein structures, especially carrying various internal repeats, a total of 23 T3Es are classified into five multigenic families of GALA, SKWP, HLK, AWR, and PopP. The construction of mutants lacking one or more T3Es reveals functional overlap among the T3Es network, and highly variable repertoires of T3Es are believed to be able to hinder the biochemical function of some T3Es in different RSSC strains (Deslandes and Genin, 2014). Our results suggested that all the T3Es members of the AWR family, but not GALA, SKWP, or HLK families, jointly contributed slightly to full virulence of OE1-1 on tobacco plants. This result is consistent with previous reports that cumulative disruption of the seven RipG (GALA) or the five RipA (AWR) or the three RipH (HLK) resulted in attenuated virulence on some hosts but had modest or no impact on others (Angot et al., 2006; Solé et al., 2012; Chen et al., 2013). For instance, T3Es members of the AWR family can induce necrotic reactions on plants and are required for full virulence of some RSSC strain (Popa et al., 2016). T3Es members of the GALA family are previously demonstrated that different GALA members display different requirements for pathogenicity on *Arabidopsis thaliana*, tomato, and eggplant (Remigi et al., 2011). We also showed that all the T3Es members of the GALA family contributed jointly in part to virulence of OE1-1 on tobacco but not on tomato plants with root-cutting inoculation (Chen et al., 2018). Mutant with deletion of all the T3Es members of 4 families of GALA, SKWP, HLK, and AWR exhibited less virulent than OE1-1 on tobacco plants (Figure 3C), probably because the six core and three extended core T3Es were deleted in the mutant. All the core and extended T3Es seem to be critical for virulence on tobacco plants in the intercellular spaces and xylem (Figures 4B,C). Intriguingly, some other T3Es besides these core and extended core T3Es contribute slightly to full virulence of OE1-1 on tobacco plants, especially in xylem vessels, indicating that different T3Es function differently in different parts of same host plants.

The RSSC possesses extremely abundant T3Es, and some of them are validated to be functionally overlapped (Deslandes and Genin, 2014). Feature of functional overlap among T3Es will inevitably hinder the individual function of single T3Es in host RSSC strains with traditional genetic disruption. Studies of single T3E function are hence usually performed by over-expressed in engineered strains. The final goal of this study is to generate a T3Es-free strain for functional studies on individual T3Es in host cells. RK7161 (OE1-1D42E) is almost T3E free that completely lost virulence on tobacco plants with leaf infiltration, and on eggplants with root-cutting inoculation. It is reported that a polymutant with deletion of 28 well-expressed T3Es in *P. syringae* pv. *tomato* DC3000 is functionally effectorless for growth in *N. benthamiana* (Sébastien et al., 2011). With the Tn*7* based chromosomal integration system, target gene of each T3Es with its native promoter can be integrated into chromosome of the T3E-free RK7161 strain at 25-bp downstream of *glmS* gene (Choi et al., 2005; Zhang et al., 2020). This sole target T3E can be thus constitutively expressed in RSSC strain and injected into host cells via the T3SS. Once this single T3E is incorporated into host cells, we can observe several phenotypes of host plant cells, which will enable primary functional study of an individual T3E in host cells.

All taken together, our results demonstrated that virulence of OE1-1 was determined with all the core and extended core T3Es. We also provided a RSSC strain lacking 60% of its T3Es, which enables primary functional studies on individual T3Es in host cells.

## Data Availability Statement

The datasets presented in this study can be found in online repositories. The names of the repository/repositories and accession number(s) can be found at: https://www.ncbi.nlm.nih.gov/nuccore/; accession numbers CP009764.1 and CP009764.1 and at: https://www.ncbi.nlm.nih.gov/nuccore/; accession numbers CP009763.1 and CP009763.1.

## Author Contributions

YZ and KO conceived and designed the experiments. NL and LC performed the experiments. AK, YH, KO, and YZ analyzed and discussed the results. YZ and NL wrote and revised the manuscript. All authors contributed to the article and approved the submitted version.

## Conflict of Interest

The authors declare that the research was conducted in the absence of any commercial or financial relationships that could be construed as a potential conflict of interest.
