# Supplementary material for: Super-Multiple Deletion Analysis of Type III Effectors in Ralstonia solanacearum OE1-1 for Full Virulence Toward Host Plants
Source: Front Microbiol. 2020 Jul 30;11:1683. doi: 10.3389/fmicb.2020.01683 (PMC7409329; doi:10.3389/fmicb.2020.01683)
Supplement: Supplementary file 1 [file Data_Sheet_1.docx]

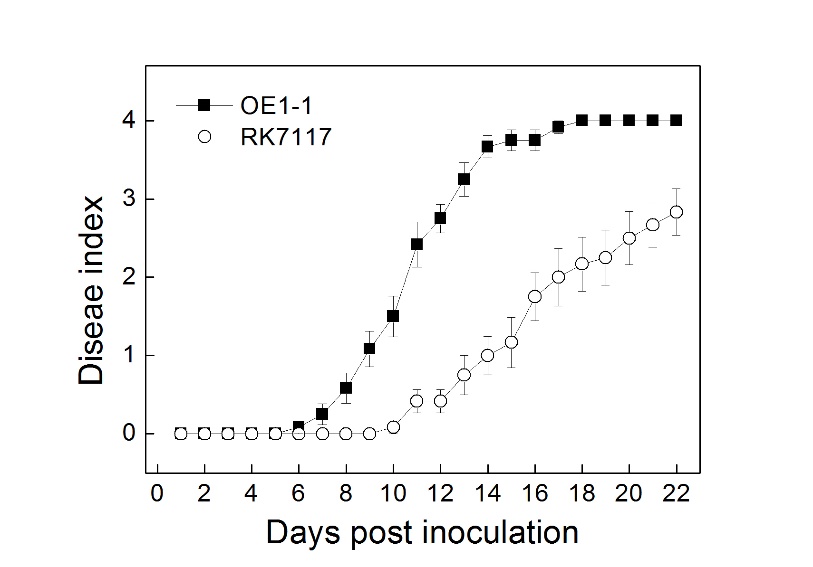


D22E


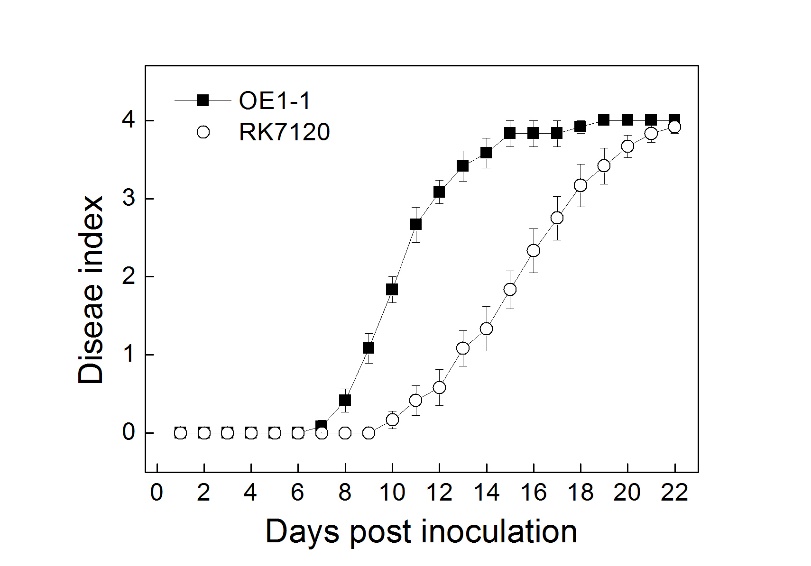


D23E


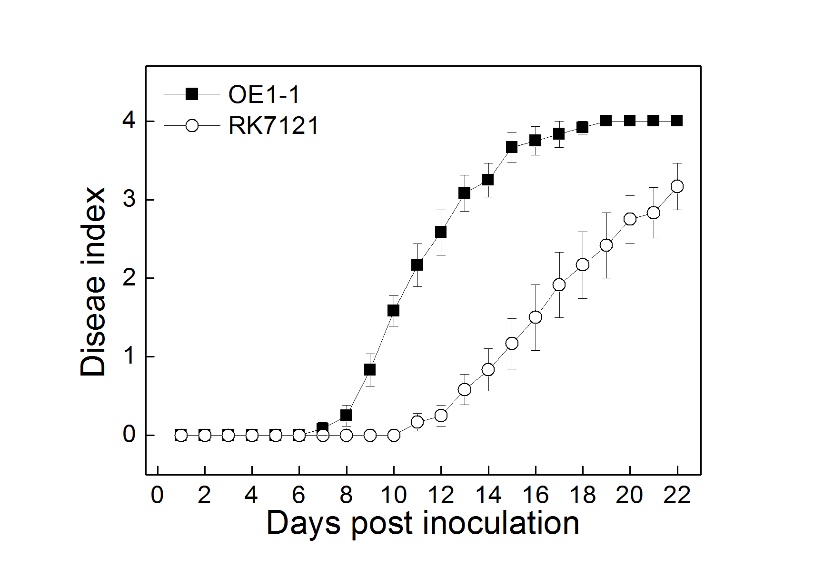


D24E


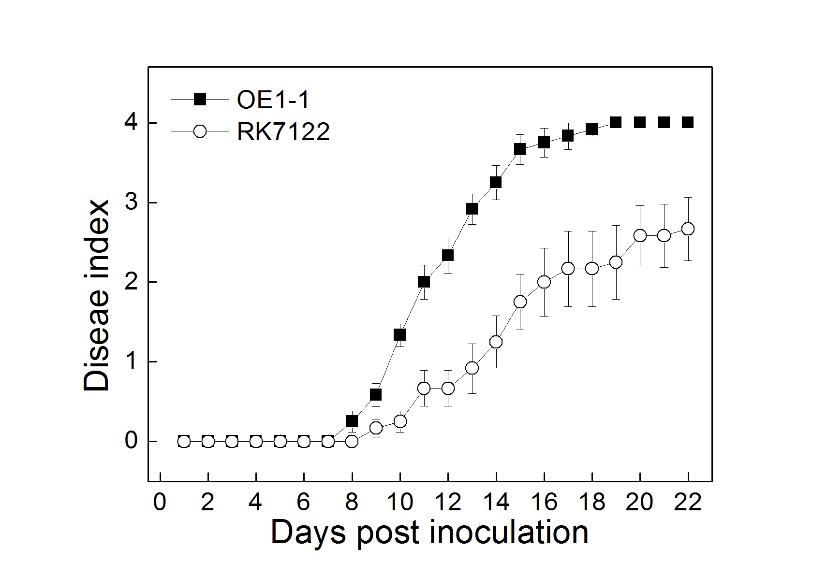


D25E


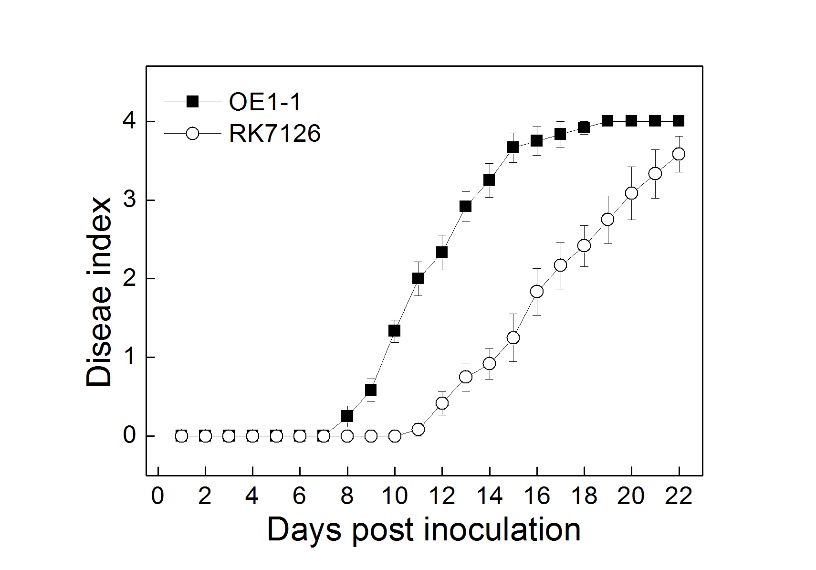


D26E


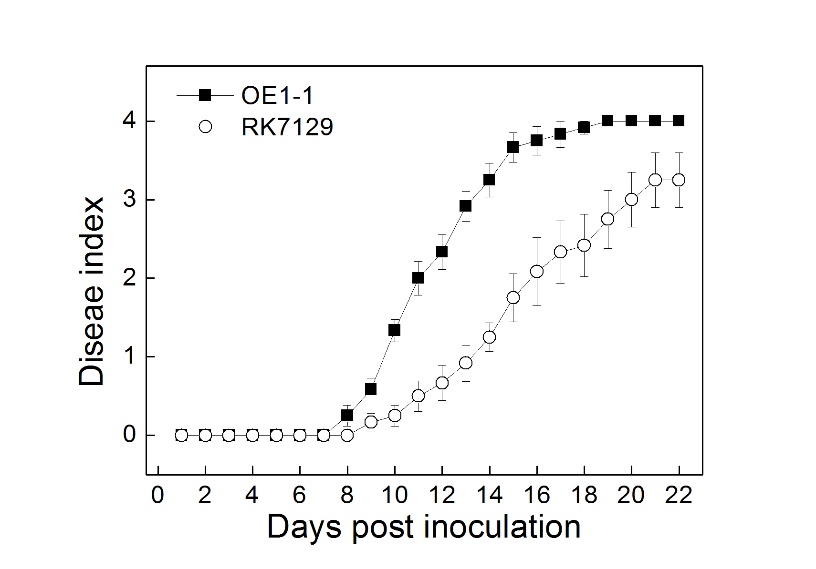


D27E


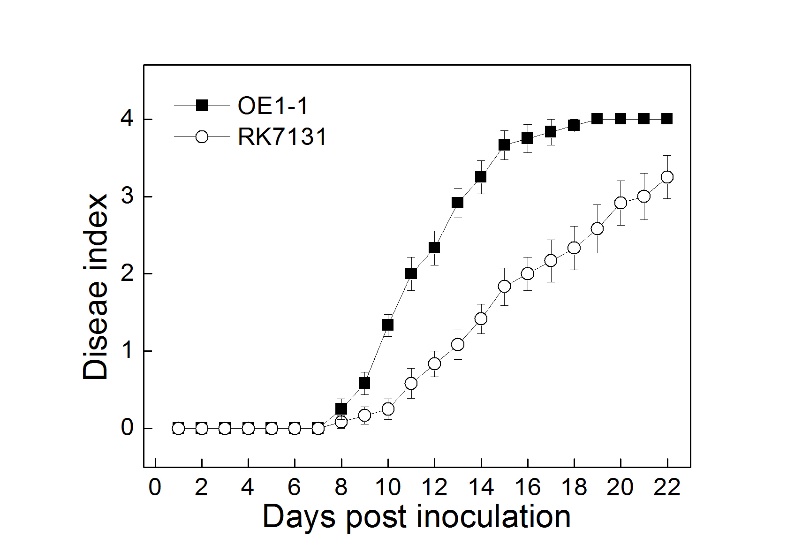


D28E


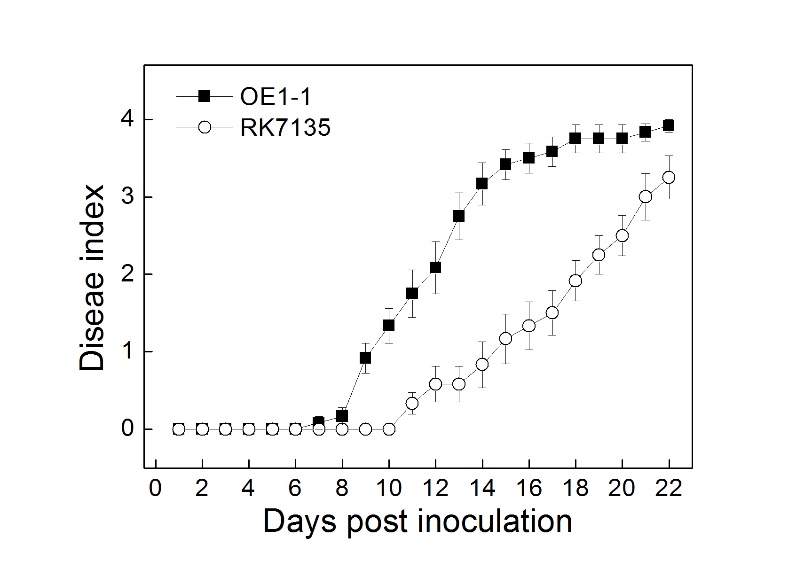


D31E


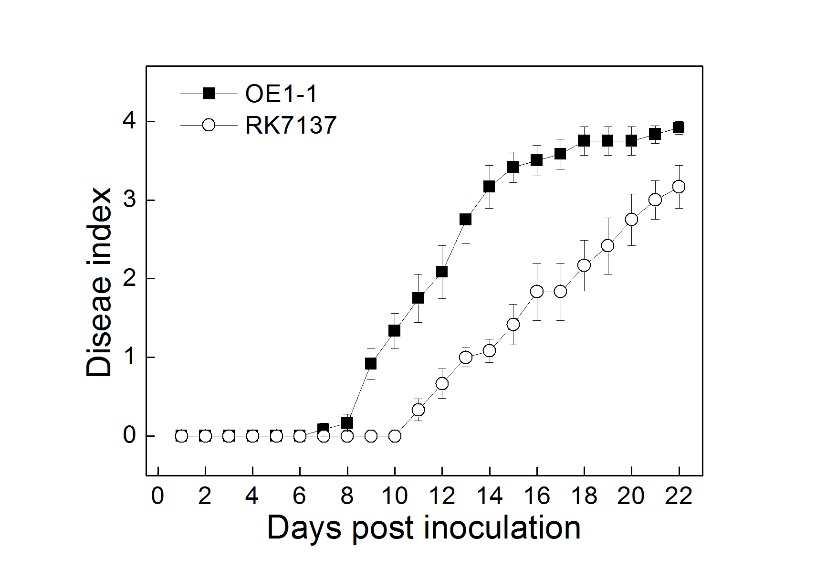


D32E


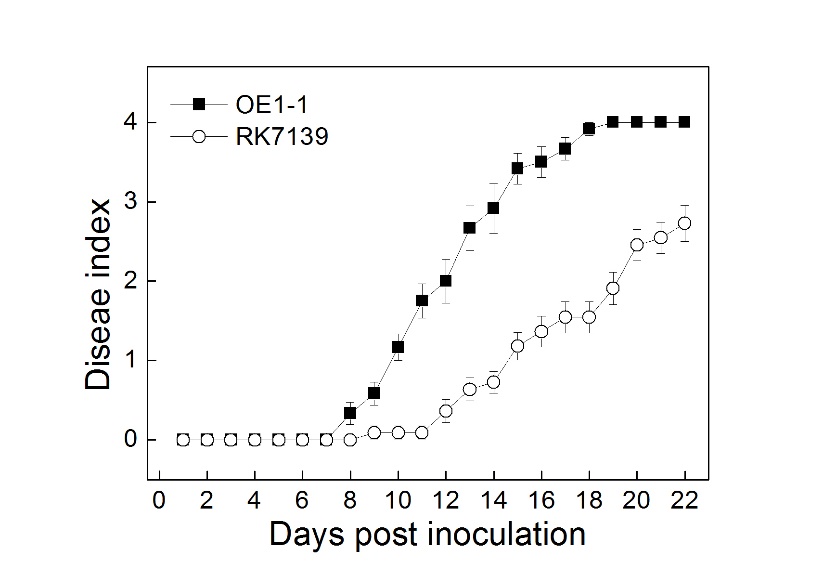


D33E


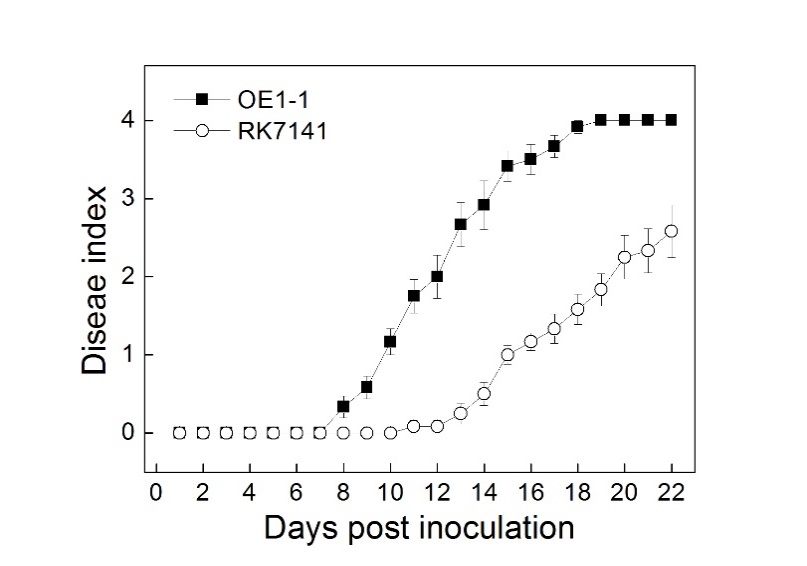


D34E


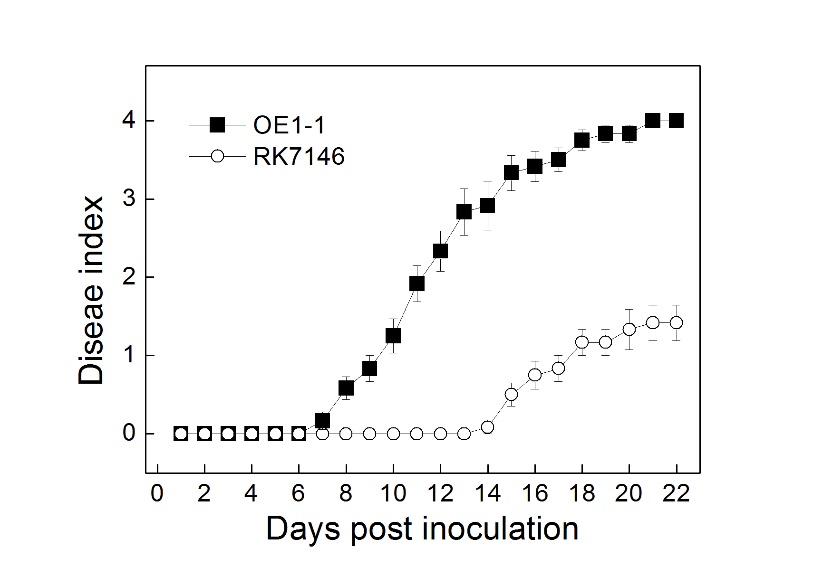


D35E


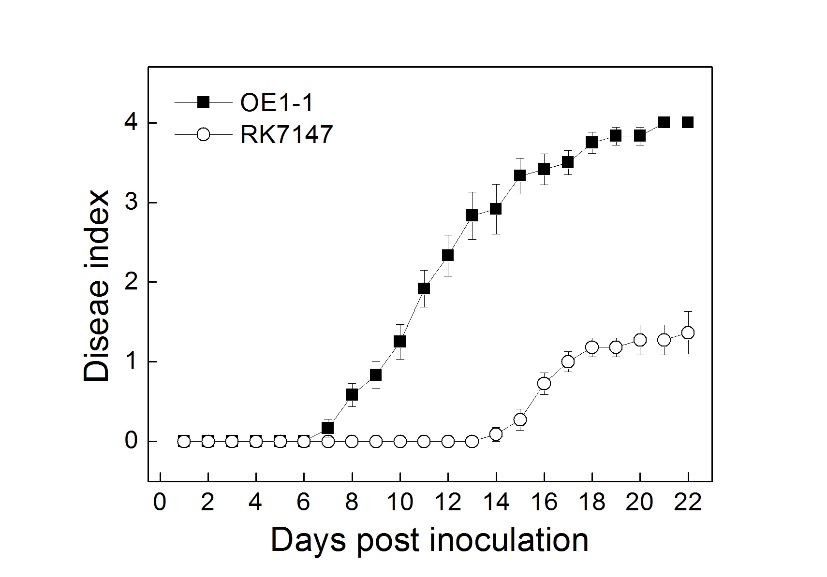


D36E

Figure S1. Disease index of different deletion mutants (delete all family members and different number of core T3Es) on tobacco (*Nicotiana benthamiana*). Number of deleted effector genes were shown in red. Tobacco leaves were hand inoculated with fresh bacterial suspensions at 10^8^ CFU mL^-1^ using a 1-mL blunt syringe. Disease symptoms were scored daily for 22 days. Plants were rated according to a scale ranging of 0 to 4 (0: no wilting; 1: 1%–25% wilting; 2: 26%–50% wilting; 3: 51%–75% wilting; 4: 76%–100% wilting). Each bacterial inoculation was tested on at least 4 plants and was repeated in triplicate. The average and standard error were calculated.
