# Supplementary material for: Super-Multiple Deletion Analysis of Type III Effectors in Ralstonia solanacearum OE1-1 for Full Virulence Toward Host Plants
Source: Front Microbiol. 2020 Jul 30;11:1683. doi: 10.3389/fmicb.2020.01683 (PMC7409329; doi:10.3389/fmicb.2020.01683)
Supplement: Supplementary file 2 [file Data_Sheet_2.docx]

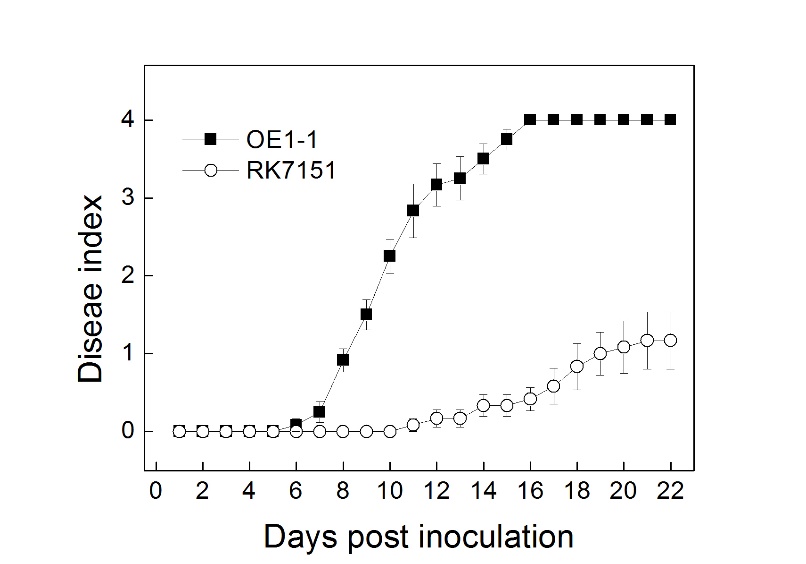


D37E


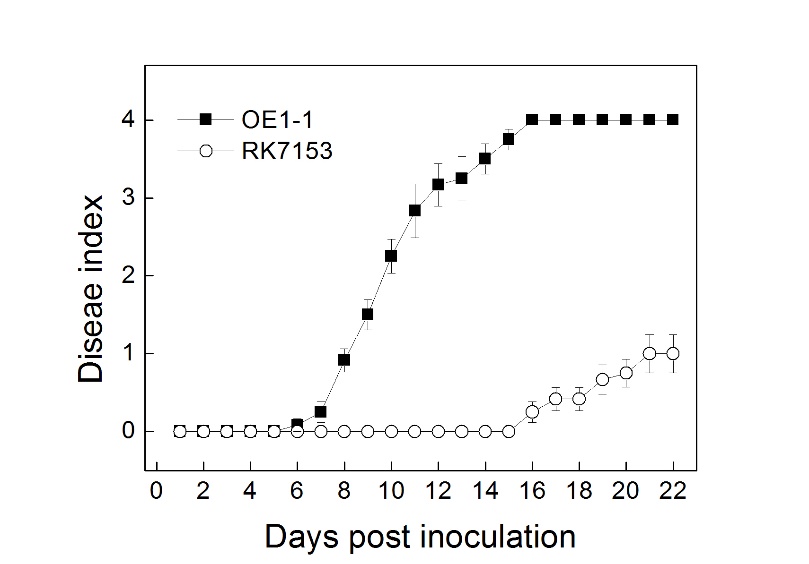


D38E


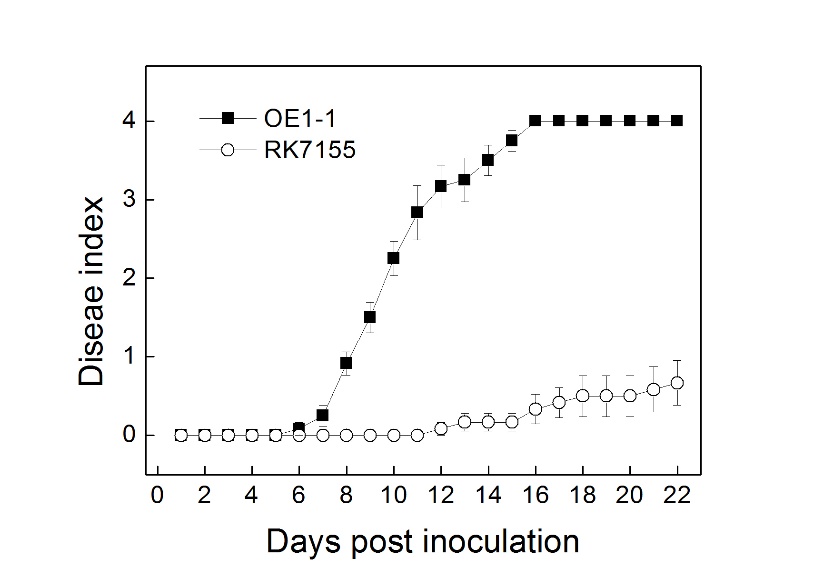


D39E


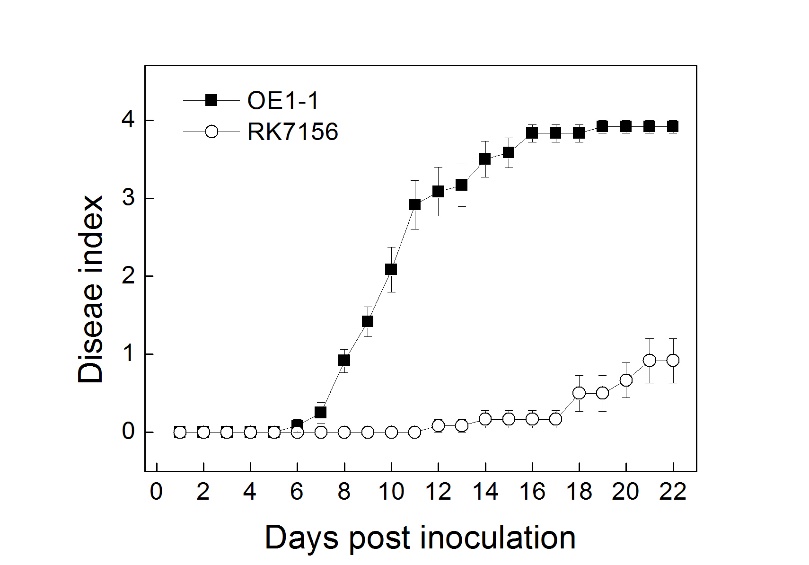


D40E


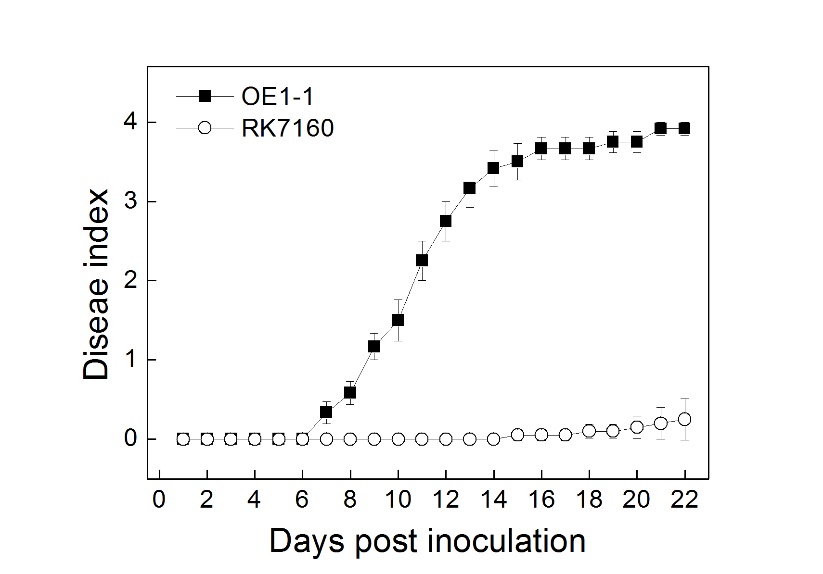


D41E


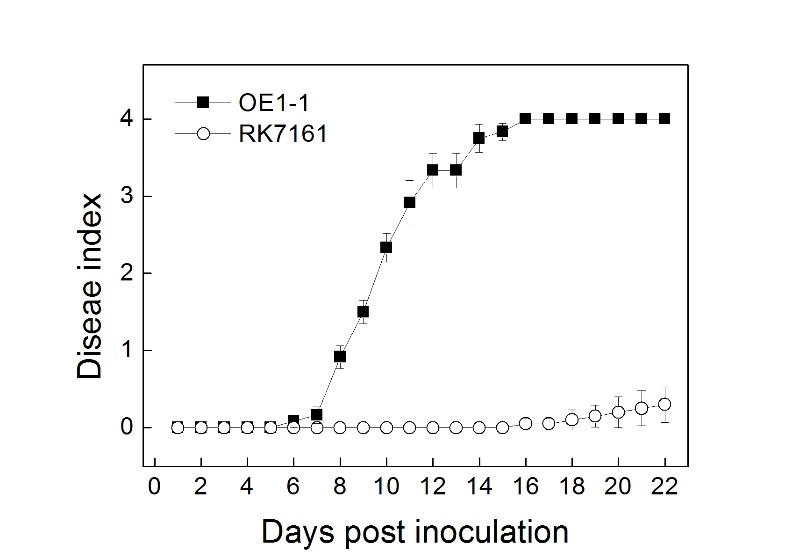


D42E

Figure S2. Disease index of different deletion mutants (delete all family members, core T3Es and different number of addtional core T3Es) on tobacco (*N. benthamiana*). Number of deleted effector genes were shown in red. Tobacco leaves were hand inoculated with fresh bacterial suspensions at 10^8^ CFU mL^-1^ using a 1-mL blunt syringe. Disease symptoms were scored daily for 22 days. Plants were rated according to a scale ranging of 0 to 4 (0: no wilting; 1: 1%–25% wilting; 2: 26%–50% wilting; 3: 51%–75% wilting; 4: 76%–100% wilting). Each bacterial inoculation was tested on at least 4 plants and was repeated in triplicate. The average and standard error were calculated.
