# Supplementary material for: Super-Multiple Deletion Analysis of Type III Effectors in Ralstonia solanacearum OE1-1 for Full Virulence Toward Host Plants
Source: Front Microbiol. 2020 Jul 30;11:1683. doi: 10.3389/fmicb.2020.01683 (PMC7409329; doi:10.3389/fmicb.2020.01683)
Supplement: Supplementary file 3 [file Data_Sheet_3.docx]

Table S1 Plasmids and primers used in this study

| effector gene | | plasmid | primer | sequence (5'- -3') |
| --- | --- | --- | --- | --- |
| unified | original |  |  |  |
| ripA1 | rsc2139 | pKC2139 | OEC2139A56 | tcgagctcggtaccccacgcaccctgttcgacacg |
|  |  |  | OEC2139B55 | ggcccgcgctgctgtccgttccaaatg |
|  |  |  | OEC2139A35 | acagcagcgcgggcctggtggcggc |
|  |  |  | OEC2139B36 | ctctagaggatcccccgccgaagatgaacttcctg |
| ripA2 | rsp0099 | pKP0099 | OEP0099A55 | tcgagctcggtaccctcgatgaaccgccgcatg |
|  |  |  | OEP0099B55 | gcgcgcctcatggcgcaaccttgaacag |
|  |  |  | OEP0099A35 | cgccatgaggcgcgcgccgccgatg |
|  |  |  | OEP0099B35 | ctctagaggatccccgacggcttctcgcgcgcg |
| ripA3 | rsp0846 | pKP0846 | OEP0846A55 | tcgagctcggtacccgccggctatgccacgctg |
|  |  |  | OEP0846B55 | gcgggaagatctgcagcgggaaccg |
|  |  |  | OEP0846A35 | tgcagatcttcccgcgaacgggcactc |
|  |  |  | OEP0846B35 | ctctagaggatccccgcgcgtgggcggcctgtg |
| ripA4 | rsp0847 | pKP0847 | OEP0847A55 | tcgagctcggtacccccggccaccccactccgc |
|  |  |  | OEP0847B56 | tggcgttcgatcgatcgtg |
|  |  |  | OEP0847A36 | atcgatcgaacgccacggaaacattcagccgacg |
|  |  |  | OEP0847B36 | ctctagaggatccccctgctgggtccgctgcac |
| ripA5 | rsp1024 | pKP1024 | OEP1024A56 | tcgagctcggtaccccaagccgcgagcatccg |
|  |  |  | OEP1024B57 | tgtatcgtgttatgtcaacaacttatgag |
|  |  |  | OEP1024A37 | acataacacgatacagcaaacgcgtc |
|  |  |  | OEP1024B36 | ctctagaggatccccgtcgccatccgggtgctg |
| ripB | rsc0245 | pKC0245 | OEC0245A55 | tcgagctcggtacccggaacgggcgctctccttg |
|  |  |  | OEC0245B55 | cgccgggagtctgacgagttgccggtc |
|  |  |  | OEC0245A35 | gtcagactcccggcgccgatccgc |
|  |  |  | OEP0245B35 | ctctagaggatcccccgatcaccaagctggccg |
| ripC1 | rsp1239 | pKP1239 | OEP1239A52 | ctgaattcccactatcgcgcgagccg |
|  |  |  | OEP1239B51 | ggatcctgcggattggatcgggctg |
|  |  |  | OEP1239A31 | ggatccccaacgccatcgcgttcatg |
|  |  |  | OEP1239B32 | ctaagcttctgctgccgatcgagctc |
| ripD | rsp0304 | pKP0304 | OEP0304A55 | tcgagctcggtacccgagctggggccgcgcaac |
|  |  |  | OEP0304B55 | ttgttgctattaaaaaatcaatccattaatc |
|  |  |  | OEP0304A35 | ttttaatagcaacaaggtcaccaacctg |
|  |  |  | OEP0304B35 | ctctagaggatccccgcccaagcgctggaagag |
| ripE1 | rsc3369 | pKC3369 | OEC3369A51 | caacaacccaccccggac |
|  |  |  | OEC3369B51 | ggatccggcaggcggctcagtgtg |
|  |  |  | OEC3369A31 | ggatcccggtcgtccgcgctcagc |
|  |  |  | OEC3369B31 | tgcgcctgttcgagctgc |
| ripG1 | rsp0914 | pKP0914 | OEP0914A51 | cactacggaaacgaggtcgcattcac |
|  |  |  | OEP0914B51 | ggatccggcagtgcgatcgtcctttctg |
|  |  |  | OEP0914A31 | ggatccgatcgccccgcgccgggcaggacg |
|  |  |  | OEP0914B31 | gccttcgcaatccgggccgtgggcgcg |
| ripG2 | rsp0672 | pKP0672 | OEP0672A51 | taatcaaaagtgactccgaagtgc |
|  |  |  | OEP0672B51 | ggatccggaagccagcccgcccaggggtg |
|  |  |  | OEP0672A31 | ggatcccgctgcgggaccgttctgcaacg |
|  |  |  | OEP0672B31 | gtggtggagcacgatgccgtgttcg |
| ripG3 | rsp0028 | pKP0028 | OEP0028A51 | gacctggatgctcgtgctgcgcg |
|  |  |  | OEP0028B51 | ggatccttccgtccgtggctccggcaaacg |
|  |  |  | OEP0028A31 | ggatccggggtgccagggcatcctcgcaac |
|  |  |  | OEP0028B31 | cgacaaattcctgatcgcctgatc |
| ripG4 | rsc1800 | pKC1800 | OEC1800A51 | tcacggcggcggatgtcgagcgcg |
|  |  |  | OEC1800B51 | ggatcccggcggtgccacccaccgcgcccg |
|  |  |  | OEC1800A31 | ggatccggcaccgccatcgggcccg |
|  |  |  | OEC1800B31 | ccggatagttgcccgcgcgc |
| ripG5 | rsc1801 | pKC1801 | OEC1801A51 | gacatcggcaacaacggcatc |
|  |  |  | OEC1801B51 | ggatccgcgccacgcccagtctgctc |
|  |  |  | OEC1801A31 | ggatccacatgccggtgagtttgccggcg |
|  |  |  | OEC1801B31 | agacccacctgctgtgggtgccg |
| ripG6 | rsc1356 | pKC1356 | OEC1356A51 | ccgagccgaagctgatcggccacg |
|  |  |  | OEC1356B51 | ggatcccgccgtctccgttacctatccatg |
|  |  |  | OEC1356A31 | ggatccatgtgacgtgtgcttgaggtgc |
|  |  |  | OEC1356B31 | atatagggatgggaagcgctg |
| ripG7 | rsc1357 | pKC1357 | OEC1357A51 | ccaaggtgctggaggccaatac |
|  |  |  | OEC1357B52 | ggatcccagctccactgcatgactatg |
|  |  |  | OEC1357A31 | ggatccggcggggcggcactgtttgccttg |
|  |  |  | OEC1357B31 | ctctctccttgtgtgatcgatccatc |
| ripH1 | rsc1386 | pKC1386 | OEC1386A55 | tcgagctcggtaccccacgccacgctggaccattg |
|  |  |  | OEC1386B55 | gcgacgcggtgttcctctatgtctgtgc |
|  |  |  | OEC1386A35 | ggaacaccgcgtcgctgtcgtgcggc |
|  |  |  | OEC1386B35 | ctctagaggatcccccgcctcggcgggatcgatg |
| ripH2 | rsp0215 | pKP0215 | OEP0215A51 | tagcaggccggggtgtcg |
|  |  |  | OEP0215B51 | ggatccggtgccctcccgatgttcg |
|  |  |  | OEP0215A31 | ggatccgcgggggctggcgtctaag |
|  |  |  | OEP0215B31 | aggctgacggtgtttgggag |
| ripH3 | rsp0160 | pKP0160 | OEP0160A51 | tgggcgtggctattaattatgatg |
|  |  |  | OEP0160B51 | ggatccggattgcccggcgctccg |
|  |  |  | OEP0160A31 | ggatcctcacgccggccgatggcg |
|  |  |  | OEP0160B31 | catggtgcccaacgccgc |
| ripI | rsc0041 | pKC0041 | OEC0041A55 | tcgagctcggtacccgcgcatgctggtgctgac |
|  |  |  | OEC0041B55 | caggtcgccgtacacctatgtcatattc |
|  |  |  | OEC0041A35 | gtgtacggcgacctgaaaggccggcctg |
|  |  |  | OEC0041B35 | ctctagaggatccccgcgagcctcaccaagcag |
| ripJ | rsc2132 | pKC2132 | OEC2132A55 | tcgagctcggtacccacgtgccatacgcatcgccgc |
|  |  |  | OEC2132B55 | cgctggggctcaactcgggctttggggc |
|  |  |  | OEC2132A35 | agttgagccccagcggggagcggcg |
|  |  |  | OEC2132B35 | ctctagaggatccccgcggcggcgctttccgag |
| ripL | rsp0193 | pKP0193 | OEP0193A55 | tcgagctcggtacccgcttgctgcaactttccaatg |
|  |  |  | OEP0193B55 | cggcccggggttcgtgctccggatg |
|  |  |  | OEP0193A35 | acgaaccccgggccgccggccgag |
|  |  |  | OEP0193B35 | ctctagaggatcccctcctgtcgcgcagcgaag |
| ripM | rsc1475 | pKC1475 | OEC1475A55 | tcgagctcggtaccccggcgctctatggttgacg |
|  |  |  | OEC1475B56 | gctcgggtcattgcggtgtcctcac |
|  |  |  | OEC1475A36 | cgcaatgacccgagcagccggaagtc |
|  |  |  | OEC1475B35 | ctctagaggatccccacgggcgagcgcgtgctg |
| ripN | rsp1130 | pKP1130 | OEP1130A55 | tcgagctcggtacccctgcgtcacaagtgctgc |
|  |  |  | OEP1130B55 | tcgccgtgtcgagagggttccgggttg |
|  |  |  | OEP1130A35 | ctctcgacacggcgagcgagaccctg |
|  |  |  | OEP1130B35 | ctctagaggatccccgttcgtactcactgggtcg |
| ripO1 | rsp0323 | pKP0323 | OEP0323A51 | cgggccggccggcaagc |
|  |  |  | OEP0323B51 | ggatccgacgcgggagccgaattg |
|  |  |  | OEP0323A31 | ggatccgcgcaagctccggcgcttg |
|  |  |  | OEP0323B31 | gatcggcgcacgggtgag |
| ripP2 | rsc0868 | pKC0868 | OEC0868A52 | gcgcccctcgcagacg |
|  |  |  | OEC0868B52 | ggatccatgttctgtgcgc |
|  |  |  | OEC0868A31 | ggatccaatcacgctgggcggtcggcg |
|  |  |  | OEC0868B31 | cggcaggcaacccttgccggtgaatc |
| ripQ | rsp1277 | pKP1277 | OEP1277A55 | tcgagctcggtaccccgaggactacctgatcctg |
|  |  |  | OEP1277B55 | ttgcgcggattcggattccgtttgc |
|  |  |  | OEP1277A35 | tccgaatccgcgcaaagccggcgagc |
|  |  |  | OEP1277B35 | ctctagaggatcccctgccggtggcggtcgccg |
| ripR | rsp1281 | pKP1281 | OEP1281A51 | ccgcttcggtttggtgtg |
|  |  |  | OEP1281B51 | ggatccaccggtcgagcgcttgc |
|  |  |  | OEP1281A31 | ggatcctctgtccgcagagcctcg |
|  |  |  | OEP1281B31 | cgcatgaagcgcatgtcg |
| ripS1 | rsc3401 | pKC3401 | OEC3401A51 | tccgcttccgatacggtgagtggtc |
|  |  |  | OEC3401B51 | ggatccggttcctgcacgctttgcattcg |
|  |  |  | OEC3401A31 | ggatccacgcggccgcgcggcggcaggc |
|  |  |  | OEC3401B31 | atcatcctgcgcagcttcctgcggc |
| ripS2 | rsp1374 | pKP1374 | OEP1374A51 | acggcggcaaactgctgcgc |
|  |  |  | OEP1374B51 | ggatccttagggttctgccgatgtttttatg |
|  |  |  | OEP1374A32 | tcggatccctgcttcagacgatgcag |
|  |  |  | OEP1374B32 | acggtggcggcataggtc |
| ripS3 | rsp0930 | pKP0930 | OEP0930A51 | tgatcccgggtgaatccattcgcggac |
|  |  |  | OEP0930B51 | ggatccccccgatccgctcccctgcgtg |
|  |  |  | OEP0930A31 | ggatcctcgtactttccgtgcaaaatc |
|  |  |  | OEP0930B31 | cagcgcatcgccgcgccgatg |
| ripS4 | rsc1839 | pKC1839 | OEC1839A51 | cgtatgaggcccgcgccg |
|  |  |  | OEC1839B51 | tctagagcggcgagggagcgacctg |
|  |  |  | OEC1839A31 | tctagacccaggcgcggcaacgggc |
|  |  |  | OEC1839B31 | gtcatcgaggtcgtcgcg |
| ripS5 | rsp0296 | pKP0296 | OEP0296A51 | ttcgccggtggccgcctt |
|  |  |  | OEP0296B52 | ctggatccaggttgtattcacaatagttc |
|  |  |  | OEP0296A31 | ggatcctgacccggtacgccggca |
|  |  |  | OEP0296B32 | ctgaattccaccaactacggcgggcag |
| ripS6 | rsc2130 | pKC2130 | OEC2130A51 | gcggcgatcattgcgtcg |
|  |  |  | OEC2130B51 | ggatccttccaaagcgttgcgtgagcg |
|  |  |  | OEC2130A31 | ggatccgcgcgtgcggccccacg |
|  |  |  | OEC2130B31 | gtatcgatgagcgcggcaag |
| ripV1 | rsc1349 | pKC1349 | OEC1349A51 | gtctgcatcggcgccgac |
|  |  |  | OEC1349B51 | ggatccctccgccagcaagccctc |
|  |  |  | OEC1349A31 | ggatcccggtccggcggtatcccg |
|  |  |  | OEC1349B31 | aagcgtgggcgatcccgc |
| ripW | rsc2775 | pKC2775 | OEC2775A55 | tcgagctcggtacccgcgggcggctcgaccatc |
|  |  |  | OEC2775B55 | ccgtcgtgactgtctcggcaagaaag |
|  |  |  | OEC2775A35 | agacagtctgctgccgctggagcgggc |
|  |  |  | OEC2775B35 | ctctagaggatccccccgacgaagtgaccgaccag |
| ripX | rsp0877 | pKP0877 | OEP0877A51 | tcaacgtgcgggcctacg |
| ripAB | rsp0876 |  | OEP0877B51 | ggatccagtgaataacctttgagggc |
| ripAC | rsp0875 |  | OEP0875A31 | ggatcctccggtgccgcatccccg |
|  |  |  | OEP0875B31 | tacgtgccggtcgccggc |
| ripY | rsc0257 | pKC0257 | OEC0257A51 | ccatgcgcgaaccggcctgcgtgc |
|  |  |  | OEC0257B51 | ggatccggcagtcctggtgagatcgggatc |
|  |  |  | OEC0257A31 | ggatccgacgccggtcgggcatcgtcatcg |
|  |  |  | OEC0257B31 | cgcggcttcggcgtgatctttcctg |
| ripZ | rsp1031 | pKP1031 | OEP1031A55 | tcgagctcggtacccgctttgccgtgtcgccgc |
|  |  |  | OEP1031B55 | cagccagctgcttgagccggtctttcag |
|  |  |  | OEP1031A35 | tcaagcagctggctggccgtcaggccggc |
|  |  |  | OEP1031B35 | ctctagaggatccccacgttttcatgggcgcagcg |
| ripAA | rsc0608 | pKC0608 | OEC0608A51 | ccgggaccttgcccatcg |
|  |  |  | OEC0608B51 | ggatcccttgatttcctgtgtttccag |
|  |  |  | OEC0608A31 | ggatcctgagtcgcccggcgccg |
|  |  |  | OEC0608B31 | tgctcgggcggcgtggtc |
| ripAD | rsp1601 | pKP1601 | OEP1601A55 | tcgagctcggtacccgtccgcggatcgtggaac |
|  |  |  | OEP1601B55 | tcgcccggcgaaccgatgcagtccg |
|  |  |  | OEP1601A35 | cggttcgccgggcgatccgacccgtc |
|  |  |  | OEP1601B35 | ctctagaggatccccgcgctcatcacgctggtc |
| ripAE | rsc0321 | pKC0321 | OEC0321A51 | cccgaccaagtccggcctgatggtc |
|  |  |  | OEC0321B51 | ggatccgatcggctccagtatcgggggcg |
|  |  |  | OEC0321A31 | ggatccatctgccggcccgtgcgagcggac |
|  |  |  | OEC0321B31 | gagcgcaaccggacaccaggcaaag |
| ripAF1 | rsp0822 | pKP0822 | OEP0822A55 | tcgagctcggtacccaagggcgtcatggcggag |
|  |  |  | OEP0822B55 | gtccacccgacgtggaagccccgtc |
|  |  |  | OEP0822A35 | ccacgtcgggtggacgcgattgcggtg |
|  |  |  | OEP0822B36 | ctctagaggatccccagctggccgccgagccg |
| ripAG | rsc0824 | pKC0824 | OEC0824A55 | tcgagctcggtacccgagcatgatcaaatcgactg |
|  |  |  | OEC0824B55 | gagagaaaagtcatctacgaatcg |
|  |  |  | OEC0824A35 | gatgacttttctctctcaggcatcgc |
|  |  |  | OEC0824B35 | ctctagaggatccccgttgcggacgtgcagag |
| ripAI | rsp0838 | pKP0838 | OEP0838A55 | tcgagctcggtaccccgccaaactgtcggaagc |
|  |  |  | OEP0838B55 | gcgcgaattctccgcgatctgttca |
|  |  |  | OEP0838A35 | gcggagaattcgcgccgattccgcg |
|  |  |  | OEP0838B35 | ctctagaggatccccacaccgagcggttcatcg |
| ripAJ | rsc2101 | pKC2101 | OEC2101A55 | tcgagctcggtacccgcttccacgacgtcgtcg |
|  |  |  | OEC2101B55 | cccgagcgggcgtcgaaacgatgaag |
|  |  |  | OEC2101A35 | cgacgcccgctcgggtgacgggctc |
|  |  |  | OEC2101B35 | ctctagaggatcccccggcagaggctgcggcag |
| ripAK | rsc2359 | pKC2359 | OEC2359A55 | tcgagctcggtacccggcggcacgccggtgatg |
|  |  |  | OEC2359B55 | catccggggtgtgtcgttccttccctg |
|  |  |  | OEC2359A35 | gacacaccccggatgcttcctcagcg |
|  |  |  | OEC2359B35 | ctctagaggatccccagcgccaaggcgaaggcg |
| ripAM | rsc3272 | pKC3272 | OEC3272A51 | gtcggagagcgacggatc |
|  |  |  | OEC3272B51 | ggatccgtcgggcggatgagcatg |
|  |  |  | OEC3272A31 | ggatccgcgctcagccgcccagc |
|  |  |  | OEC3272B31 | gaactggcgacgcggacg |
| ripAN | rsp0845 | pKP0845 | OEP0845A55 | tcgagctcggtacccgccgctgcggaacctggc |
|  |  |  | OEP0845B55 | gggcgggtgggagtctccgggttgggc |
|  |  |  | OEP0845A35 | gactcccacccgccccggcaggc |
|  |  |  | OEP0845B35 | ctctagaggatcccctgctcggcgaagggcatg |
| ripAO | rsp0879 | pKP0879 | OEP0879A55 | tcgagctcggtaccctgcgcggacatcttgccg |
|  |  |  | OEP0879B55 | gtaccgcctggcggctccctcgcg |
|  |  |  | OEP0879A35 | gccgccaggcggtacccccagtcaga |
|  |  |  | OEP0879B35 | ctctagaggatccccccgacatttcgcgcggcg |
| ripAQ | rsp0885 | pKP0885 | OEP0885A56 | tcgagctcggtacccgcgcgttcgtcgccgacg |
|  |  |  | OEP0885B56 | gaaccgcggcgatgcacctcatgtc |
|  |  |  | OEP0885A36 | gcatcgccgcggttccttgacgcacg |
|  |  |  | OEP0885B37 | ctctagaggatcccccctcggccgacaagcgtg |
| ripAR | rsp1236 | pKP1236 | OEP1236A55 | tcgagctcggtacccgccatgaagagcttcgcg |
|  |  |  | OEP1236B55 | gaccgcttggtgtcggtgtcggatc |
|  |  |  | OEP1236A35 | cgacaccaagcggtctgcccgaagcg |
|  |  |  | OEP1236B35 | ctctagaggatcccctacctggacgggcgggag |
| ripAS | rsp1384 | pKP1384 | OEP1384A51 | tcgatgtagcccgtggccgggatg |
|  |  |  | OEP1384B51 | ggatccggcctggatggaccggctcactcg |
|  |  |  | OEP1384A31 | ggatccccggcgcctcgtcgtccggacgc |
|  |  |  | OEP1384B31 | atctacctgccgcccgccgaggcg |
| ripAT | rsp1388 | pKP1388 | OEP1388A55 | tcgagctcggtaccccatcgcagcgcgcggccg |
|  |  |  | OEP1388B55 | gccggtgggctccgcgggcagcgtc |
|  |  |  | OEP1388A35 | gcggagcccaccggccgcagccctac |
|  |  |  | OEP1388B35 | ctctagaggatcccccagctgcgcgagcgacag |
| ripAU | rsp1460 | pKP1460 | OEP1460A55 | tcgagctcggtacccgcaagatagggcgcgctc |
|  |  |  | OEP1460B55 | gtgcccgggcggcccgggacagatc |
|  |  |  | OEP1460A35 | gggccgcccgggcacgccggttgacg |
|  |  |  | OEP1460B35 | ctctagaggatccccgtcggcgcaagcgcgcag |
| ripAV | rsp0732 | pKP0732 | OEP0732A51 | cccgagggcgaagac |
|  |  |  | OEP0732B51 | ggatccggtggtcgctacgtcctg |
|  |  |  | OEP0732A31 | ggatccgcggtgttcatgctgtgc |
|  |  |  | OEP0732B31 | taccgcctatgctcgttg |
| ripAW | rsp1475 | pKP1475 | OEP1475A55 | tcgagctcggtacccccgatcgctgtggccagc |
|  |  |  | OEP1475B55 | gccccgggggctcgcgcggcgatg |
|  |  |  | OEP1475A35 | gcgagcccccggggcgcatcggtg |
|  |  |  | OEP1475B35 | ctctagaggatccccgggctgatccgtctcctg |
| ripAX1 | rsc3290 | pKC3290 | OEC3290A51 | ttggtgaaggtgacggcggcgatg |
|  |  |  | OEC3290B51 | ggatcccggcgtgttgaggtgttgttggac |
|  |  |  | OEC3290A31 | ggatccgacgacatccgccggcaag |
|  |  |  | OEC3290B31 | ggcgatcttcccggtgcagcttggc |
| ripAY | rsp1022 | pKP1022 | OEP1022A55 | tcgagctcggtaccccctgcgacggatttgcgc |
|  |  |  | OEP1022B55 | agtggcgggctggcaccgtcgatcg |
|  |  |  | OEP1022A35 | tgccagcccgccactcgccattctctc |
|  |  |  | OEP1022B35 | ctctagaggatccccacacttaatggcaagaaatcg |
| ripAZ1 | rsp1582 | pKP1582 | OEP1582A55 | tcgagctcggtacccgaccctgccgtccgagtg |
|  |  |  | OEP1582B55 | ggaatccggaccggacataaggaattg |
|  |  |  | OEP1582A35 | tccggtccggattccatcgccctcaag |
|  |  |  | OEP1582B35 | ctctagaggatcccccgtggaccgcacctgcag |
| ripBO | rsc3174 | pKC3174 | OEC3174A51 | gtgtgccgtccgctaccgctacgtg |
|  |  |  | OEC3174B51 | ggatcccgggtacgggtcggcaacggcg |
|  |  |  | OEC3174A31 | ggatccaagaatgtctccgtatcccatgtg |
|  |  |  | OEC3174B31 | ggaatgcccagcgccagctcgtg |
| ripTAL1 | rsc1815 | pKC1815 | OEC1815A51 | aaggtcacgtcgatattgatg |
|  |  |  | OEC1815B51 | ggatcctgcatcctgattcctaaggat |
|  |  |  | OEC1815A31 | ggatcccagtcgccaacgttgcgc |
|  |  |  | OEC1815B31 | ccggacggtgggcgacac |
| ripTPS | rsp0731 | pKP0731 | OEP0731A51 | cgcccggcgcctgcagtc |
|  |  |  | OEP0731B51 | ggatcctgcgtgttcgtattgcctttg |
|  |  |  | OEP0731A31 | ggatccccggccgtgcccgcgc |
|  |  |  | OEP0731B31 | ctcgggcggcgatccatc |
